# The potential of IFN-λ, IL-32γ, IL-6, and IL-22 as safeguards against human viruses: a systematic review and a meta-analysis

**DOI:** 10.3389/fimmu.2024.1303115

**Published:** 2024-02-14

**Authors:** Areej A. Sattar, Ariba Qaiser, Hina Kausar, Sarah Aqil, Rida Mudassar, Sobia Manzoor, Javed Ashraf

**Affiliations:** ^1^ Molecular Virology Lab, Atta-Ur-Rahman School of Applied Biosciences (ASAB), National University of Science & Technology (NUST), Islamabad, Pakistan; ^2^ Department of Community Dentistry, Islamabad Medical and Dental College (IMDC), Islamabad, Pakistan; ^3^ Institute of Dentistry, University of Eastern Finland (UEF), Kuopio, Finland

**Keywords:** interleukin-6, interleukin-22, interleukin-32 gamma, interferon-lambda, antiviral

## Abstract

Many studies have investigated the antiviral activity of cytokines, including interleukin-6 (IL-6), interleukin-22 (IL-22), interleukin-32 gamma (IL-32γ), and interferon-lambda (IFN-λ) in diverse populations. This study aims to evaluate the role of these cytokines in inhibition of various human and animal viruses when administered exogenously. A comprehensive meta-analysis and systematic review were conducted on all the relevant studies from three databases. Standard mean differences (SMDs) of overall viral inhibition were used to generate the difference in the antiviral efficacy of these cytokines between control and experimental groups. A total of 4,618 abstracts for IL-6, 3,517 abstracts for IL-22, 2,160 abstracts for IL-32γ, and 1,026 abstracts for IFN-λ were identified, and 7, 4, 8, and 35 studies were included, respectively, for each cytokine. IFN-λ (SMD = 0.9540; 95% CI: 0.69–0.22) and IL-32γ (SMD = 0.459; 95% CI: 0.02–0.90) showed the highest influence followed by IL-6 (SMD = 0.456; CI: −0.04–0.95) and IL-22 (SMD = 0.244; 95% CI: −0.33–0.81). None of the cytokines represented heterogeneity (tau² > 0), but only IFN-λ indicated the funnel plot asymmetry (p = 0.0097). Results also indicated that IFN-λ and IL-32γ are more potent antivirals than IL-6 and IL-22. The collective findings of this study emphasize that exogenously administered pro-inflammatory cytokines, specifically IFN-λ and IL-32, exhibit a significant antiviral activity, thereby underscoring them as potent antiviral agents. Nonetheless, additional research is required to ascertain their clinical utility and potential for integration into combinatorial therapeutic regimens against viral infections.

## Introduction

Viruses cause infections in human beings including adults and children. Behind most of the infections, the culprits are viruses and are far more frequent than bacterial, fungal, and other infections (1). On average, children suffer from viral respiratory infection around 10 times a year during early childhood (2). The spectrum of afflictions caused by viruses’ range from benign illnesses like common cold, flu (3), and warts (4) to severe diseases like acquired immune deficiency syndrome (5), coronavirus disease 2019 (COVID-19) (6), Influenza (7), and Ebola (8). Viruses are mainly composed of genetic information enclosed in proteins with keys to unlock the gateways of a specific or wide range of host cells. Researchers have so far identified approximately 270 species of viruses that are pathogenic to humans, but many are undiscovered (9).

In terms of their pathogenicity, viruses follow both acute and chronic courses. Manifestations of acute viral infections range from mild symptoms like cough, runny nose, and mild fever due to Rhinovirus, to severe morbidities like hemorrhagic fever, bleeding, persistent pain in the chest, shortness of breath, caused by dengue virus, and coronavirus 2. On the other hand, there are viruses capable of integrating themselves into the human genome and staying dormant, without showing any signs and symptoms for a while, leading to a chronic infection or carrier state. Example of these chronic viral pathogens include Epstein–Barr virus (10) and cytomegalovirus (CMV) (11). Whereas, some continue to express their genes at low concentrations and suddenly rise, e.g., human papillomavirus, hepatitis B virus (HBV), and hepatitis C virus (HCV) (12).

Antibiotics are no solutions to rescue human beings from viruses (13) although some antiviral drugs are available for the cure. Food and Drug Administration (FDA) has approved mostly small molecules and some large molecules such as oligonucleotides, interferons, and monoclonal antibodies against viral infections (14). Most of the antiviral drugs are inhibitors or base analogs to inhibit viral replication and gene expression (15). Despite the effectiveness of the approved antiviral drugs, there are several viral infections that cannot be cured with them, for example, influenza has developed the resistance against its PA inhibitor baloxavir marboxil (16), as well as ganciclovar resistance in transplant patients against CMV (17). Moreover, no or poor response, relapse, drug resistance mutation, narrow spectrum targets, and harmful side effects demand alternative strategies (18).

The immune system provides natural defense against viruses by activating innate (19) and adaptive (20) mechanisms, especially in eliminating acute viral infections, making them last for a week approximately (21). However, during chronic viral infections, the persistence of the virus results in desensitization and exhaustion of the immune system. The combination of antigen-presenting cells, phagocytes, and cytokines; the bridge between the cell-mediated and humoral immune system; and lymphocytes form an army to fight against the virus (22). Despite the powerful defense mechanism, viruses have developed different escape strategies by constantly evolving themselves via antigenic shift and drift.

Immunotherapy is an advanced field to accelerate the natural defense mechanism against viruses in which scientists have exploited different components of the immune system (23). Cytokine immunotherapy involves the exogenous intake of either cytokine expression vector or recombinant cytokines, either *in vitro*, *in vivo*, or both. These cytokines have been observed in inducing an effective antiviral state. Pro-inflammatory cytokines like type 1 interferons (IFNs), interleukin-1β (IL-1β), interleukin-6 (IL-6), interleukin-18 (IL-18), and tumor necrosis factor–α (TNF-α) can enhance viral neutralization, reduction in viral replication, and apoptosis of viral infected cells (24). The exact mechanism with most cytokines protects cells or a living system from viruses is yet to be fully understood. Growing evidence reports that they enhance antigen presentation by Major Histocompatibility Complex (MHC) molecules and stimulate cell-mediated immune cells such as natural killer cells, cytotoxic T cells, and CD8+ T cells. A direct antiviral state can also be achieved by some cytokines that enhance virus killing and virus-infected cell apoptosis (25).

There are insufficient data available on other pro-inflammatory cytokines that are used, but IFN-λ, IL-32γ, IL-6, and IL-22 have been extensively reviewed to determine their exogenous antiviral activity against different viruses. In order to obtain the valuable significance of such experimental projects, here, we performed a systematic review and meta-analysis of these studies. Pooling data from several studies to make a literature review sometimes leads to biasness of the subject. This meta-analysis presents a systematic and absolute conclusion on the antiviral effect of the mentioned cytokines through statistical analysis. As commercially available FDA-approved antiviral drugs are not effective against all viral diseases, the administration of these cytokines presents an excellent alternative medicine. The objective of this meta-analysis is to evaluate whether these selected cytokines can be either an alternative or combinatorial medicine.

## Methods

### Study design

This systematic review and meta-analysis was carried out in the light of Cochrane Handbook principles (26) and Preferred Reporting Items for Systematic Reviews and Meta-Analysis (27). Four separate sets of analyses were used to investigate the antiviral activity of each interleukin.

### Study selection strategy

The search for the relevant studies began through major databases including Google Scholar, PubMed, and ScienceDirect. Only research papers from 1 January 2000 to 1 January 2023 were opted for and searched with the specific terms using advanced setting. The terms were decided with mutual agreement by all the authors as the chances were high to locate the required information (find below in inclusion criteria) in the articles mentioned with them. Separate searches were carried out using specific terms in the title of articles, “interleukin-28, interleukin-29, and interferon-lambda (as it is called with all the three names), and interleukin-22, interleukin 6, and interleukin-32” or their abbreviations “IL- 28, IL-29, and IFN-λ, and IL-22, IL-6, and IL-32” with “antiviral” or “virus” for each cytokine individually.

Three authors (Hina Kausar, Sarah Aqil, and R. Mudassir) were assigned to review all the titles, abstracts, and full texts to evaluate whether the selected articles meet the inclusion criteria for data extraction. In case of any disagreement about the article to be included or excluded, the corresponding author (Sobia Manzoor) reviewed the study and made the decision.

### Inclusion criteria

Research articles from the year 2000 to 2023 were included to report recent progress in the relevant topic with experimental studies *in vitro* (primary and secondary cell cultures) or *in vivo* (animal models; mice, etc.) were included. The aim of the study could only be accomplished where the selected research must contain the experiments where cell culture(s) or an animal model were infected with any human virus and treated with any of the selected cytokines. Therefore, the inclusion of only *in vitro* or *in vivo* studies was crucial. Only the experimental studies utilizing exogenous IFN-λ, IL-6, IL-22, and IL-32γ (either recombinant proteins or expression vectors), treatment of pre– or post–human viral infections. Viral titers (quantity of viral DNA/RNA or/and viral proteins) must be mentioned as mean of the results with SEM (standard error of mean).

### Exclusion criteria

Review articles, retrospective studies, and articles carrying endogenous antiviral effect of cytokines and animal and plant viruses were not included. The animal and plant systems are quite different from the human system in terms of the receptors that bind to viruses and also the responses that they generate during a viral infection; therefore, they should be separately reviewed and must not be included in this review. Articles following synergistic roles of selected cytokines with other compounds were also excluded. If other compounds are also used in combination with one of the selected cytokines to evaluate their antiviral potential, then it is difficult to report whether which one of these was truly responsible to generate that response. In addition, if the reduction in viral titer is due to their synergy, then the aim of the review is not justified, which is attributed to only the IFN-λ, IL-6, IL-22, and IL-32γ.

### Data extraction

Articles that were present in duplication were assessed by Zotero to use it only once (28). Separate data sheets were created for all of the four cytokines. They included different columns, study name and year, name of viruses, name of cell lines/animal model, pre- or post-infection treatment with cytokines, cytokine dose, techniques for measuring viral titter, mean viral titers in the control (untreated), and cytokine-treated group and number of repetition of experiments. The mean viral titers were measured through the graphs present in the studies. Studies with multiple viruses were divided into separate rows. Standard deviation was calculated with the given mean and number of replicates.

### Statistical analysis

The analysis was carried out on Jamovi version 2.3.28 (29), using the standardized mean difference as the outcome measure (30). The statistical significance was measured through z-test. A random-effects model was fitted to the data. The amount of heterogeneity (i.e., tau^2^) was estimated using restricted maximum likelihood estimator (31). In addition, to estimate of tau^2^, the Q-test for heterogeneity (32) and the I^2^ statistics are reported. In case any amount of heterogeneity detected (i.e., tau^2^ > 0, regardless of the results of Q-test), a prediction interval for the true outcome was also provided. Studentized residuals and Cook’s distances were used to examine whether studies may be outliers and/or influential in the context of our specified model. Studies with studentized residual larger than 100 × (1 − 0.05/2 × k)th percentile of a standard normal distribution were considered potential outliers (i.e., using Bonferroni correction with two-sided alpha = 0.05 for K studies included in the meta-analysis). Studies with a Cook’s distance larger than the median plus six times the interquartile range of the Cook’s distances were considered to be influential. The rank correlation test and the regression test, using the standard error of the observed outcomes as the predictor, are used to check funnel plot asymmetry (33).

## Results

**Figure 1:**
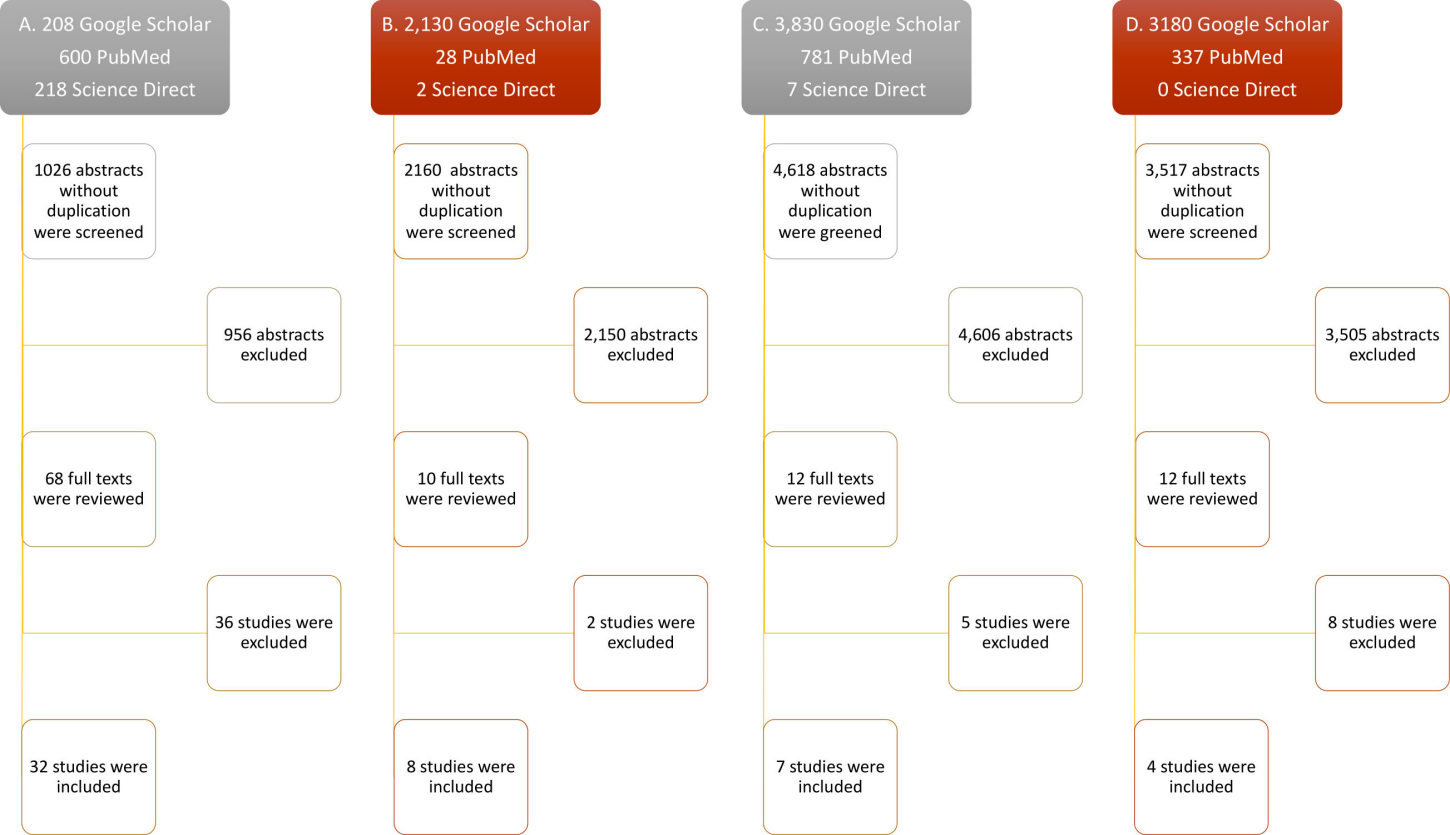
Flow diagram of study selection. Only research articles’ titles with the keywords (mentioned in methods) were selected for abstract reading. The relevant abstracts were included after duplication removal. Accessible full texts were screened and included. Full texts lacking exogenous cytokine treatment against viruses, mean viral titters with SEM, and number of replicates were excluded. **(A–D)** Study selection steps of IL-32, IFN-λ, IL-6, and IL-22, respectively.

The advanced search on each data base revealed 1,026 abstracts for IFN-λ, 2,160 for IL-32γ, 4,618 for IL-6, and 3,517 for IL-22, collectively. A total of 956 abstracts for IFN-λ, 2,150 for IL-32γ, 4,606 for IL-6, and 3,505 for IL-22 were excluded due to the absence of exogenous treatment of these cytokines to evaluate their antiviral potential. Most of the excluded articles were about innate antiviral response of cytokines, especially in the case of IL-6, and others did not include the information about viral quantification or number of antiviral experiments. Subsequently, a total of 36 full texts for IFN-λ, 2 for IL-32γ, 5 for IL-6, and 8 for IL-22 were excluded. The included studies contained the viral titers and number of experiments, following the inclusion criteria. The greatest number of studies found was 32 for IFN-λ, then 8 for IL-32γ, 7 for IL-6, and 4 for IL-22. The inclusion and exclusion breakdown has been shown in **Figure 1**.

**Tables 1**–**4** represent systematic reviews of each of the cytokines with the extracted data that are required according to the inclusion criteria. Nearly all the most prevalent critical viral disease causative agents were included in the studies to observe the antiviral response of cytokines treatment on them. The cytokines were used either in the form of recombinant proteins, plasmids, or viral vectors expressing them. The experiments were conducted mostly on primary or secondary cell cultures and some on animal models. The treatments were either before or after viral infection, and, later, the viral titers were determined through various methods, out of which quantitative real-time polymerase chain reaction (qRT-PCR) being the most commonly used.

**Table 1:** Characteristics of studies included on IFN-λ.

Study	Virus	Population	Dose (ng/mL)	Pre-infection Treatment with cytokine	Co/Post-infection treatment with cytokine	Method of evaluation of viral titer	No. of replicates
Ank et al., 2006 (34)	EMCV, HSV2, and LCMV	HepG2	MOI of 0.15 MOI of 0.015	Yes	Yes	qRT-PCR	5–7
Shindo et al., 2013 (35)	HCV	OR6 and Huh7.5.1	0.0, 0.01, 0.1, 1, 10, 100, and 1,000	Yes	Yes	Luciferase activity CLEIA qRT-PCR	3
Brand et al., 2005 (36)	HCMV	HCT116 cells	10 100	Yes	Yes	qRT-PCR ELISA	2
Wang et al., 2015 (37)	HIV	Monocyte- derived macrophages	10 100	Yes	Yes	qRT-PCR	3
Plotnikova et al., 2021 (38)	IAV ADV	A549 cells and Vero cells	10, 100, or 500	Yes	Yes	qRT-PCR In-cell ELISA	3
Caine et al., 2019 (31)	ZIKV	HVECs and HCECs	100	Yes	No	qRT-PCR	4
Hong et al., 2007 (32)	HBV	WT10 and PEB8	1,000	No	Yes	Southern blot qRT-PCR	3
Chong et al., 2022 (33)	SARS-CoV-2 (Omicron)	K18h-ACE2 transgenic mice	2,000	Yes	Yes	Plaque assay	8
J. Li et al., 2011 (39)	HSV-1	Primary human astrocytes and primary human neurons	100	Yes	No	Immunofluorescence assay qRT-PCR	3
Medaglia et al., 2021 (40)	Influenza A (H1N1) pdm09	MDCK and Calu-3	12.5–100	Yes	Yes	qRT-PCR Virus yield reduction assay	2
Su et al., 2018 (41)	HIV	Purified human monocytes	100, 250, and 500	Yes	Yes	HIV RT assay qRT-PCR	3
Madonov et al., 2021 (42)	SARS- CoV-2	VeroE6	0.16–42,500	Yes	No	MTT cell viability assay	3
Lopušná et al., 2014 (43)	HSV-1 (KOS and ANG-path strain)	Vero E6 cells	10, 15, 20, 25, 30, 35, 40, 70, 100, and 130	Yes	No	Plaque assay qRT-PCR	2
M. Hong et al., 2016 (44)	HCV WNV	Huh7 cells	100	NA	NA	Renilla luciferase reporter activity qRT-PCR	3
Lukacikova et al., 2015 (45)	LCMV	A549 cells	20	Yes	NA	qRT-PCR Western blot	2
Ma et al., 2009 (46)	WNV	Huh7.5 and Hela cells	0.8, 4, 20, 100, and 500	Yes	Yes	Renilla luciferase assay qRT-PCR	3
X. Li et al., 2017 (47)	HCV	Huh7	100	No	Yes	qRT-PCR Western blot	3
Mallampalli et al., 2021 (48)	Influenza CA09 and Influenza PR8	Macrophages	50	Yes	No	qRT-PCR	3
Y. Li et al., 2020 (49)	RABV	NA cells or Vero cells	10 or 1,000	No	Yes	qRT-PCR	3
Hamming et al., 2013 (50)	HCV, HCoV- 229E and MERS-CoV	Huh7, Hepg2 and human HAE cultures	0.0001–1,000	No	Yes	Renilla luciferase assay qRT-PCR	3
Hou et al., 2009 (51)	HIV-1	Macrophages	10 to 1,000	Yes	No	qRT-PCR	3
Svetlikova et al., 2010 (52)	IAV	MDCK, HeLa, A549, and Vero cells	10 to 40	Yes	No	Plaque assay	2
Yamauchi et al., 2016 (53)	HCV	STAT1 knockout cells and STAT2 knockout cells	1,000 U/mL	No	Yes	qRT-PCR	3
Liu et al., 2012 (54)	HIV	Macrophages	12.5, 25, 50, and 100	Yes	Yes	qRT-PCR Indirect immunofluorescence assay	3
Q. Chen et al., 2021 (55)	HCV	Huh7	1 10	No	Yes	Luciferase assay	4
Busnadiego et al., 2020 (56)	SARS CoV-2	Calu-3	1,000	Yes	No	Plaque assay	3
Felgenhauer et al., 2020 (57)	SARS CoV-1 and SARS CoV-2	Calu-4	10	Yes	No	Plaque assay	3
Makjaroen et al., 2018 (58)	HBV	HepG2.2.15 cells	1,000 ng/mL	No	Yes	qRT-PCR	3
Sauerhering et al., 2017 (59)	NiV	Differentiated HBEpCs from human donor	10 ng/mL	Yes	No	qRT-PCR	3
Z. Li et al.,2017 (60)	HSV-2	End1/E6E7 cells	100 ng/mL	yes	no	qRT-PCR	3

EMCV, encephalomyocarditis virus; HSV2, herpes simplex virus; LCMV, lymphocytic choriomeningitis virus; HCV, hepatitis C virus; HCMV, human cytomegalovirus; HIV, human immune deficiency virus; IAV, influenza A virus; ADV, adenovirus; ZIKV, Zika virus; HBV, hepatitis B virus; SARS-CoV-2, severe acute respiratory syndrome coronavirus 2; WNV, West Nile virus; RABV, rabies virus; HCoV, human coronavirus; MERS-CoV, Middle East respiratory syndrome coronavirus; NiV, Nipah virus; MOI, Multiplicity of Infection; HBEpCs, human bronchial epithelial cells; qRT-PCR, quantitative real-time polymerase chain reaction; ELISA, enzyme-linked immunosorbent assay.

**Table 2:** Characteristics of studies included on IL-32γ.

Study	Virus	Population	Dose (ng/mL)	Pre-infection treatment with cytokine	Co/Post-infection treatment with cytokine	Method of evaluation of viral titer	No. of replicates
Y. Li et al., 2013 (61)	HBV EV71 HIV HCV	HepG2.2.1.5 cells, L02 cells, Huh7 cells, Hep3B cells, and PBMCs	5	No	Yes	qRT-PCR ELISA	6
W. Li et al., 2010 (62)	IAV	MDCK cells	10 20 40	Yes	No	Hemagglutination assay qRT-PCR	6
Nold et al., 2008 (63)	HIV	PBMC and U1 macrophage cell line	1 10	Yes	No	ECL and ELISA	10
Rasool et al., 2008 (64)	HIV	HEK 293T cells and Jurkat T cells	N/A	Yes	Yes	HIV LTR: luciferase assay HIV p24: ELISA	3
Zepp et al., 2011 (65)	VSV HSV-2	WISH cells and Vero cells	VSV: 0.5, 1, 5, 10, 20, and 50 HSV2: 1, 5, 10, 20, 50, and 100	Yes	No	Crystal Violet staining and LDH assay for cell viability	8
Mesquita et al., 2017 (66)	HIV	HIV-infected CD4+ cells	100	No	Yes	qRT-PCR	12
Kim et al., 2018 (67)	HBV	Huh7 BALB/C mice	IL-32–expressing plasmid: 0.1, 0.5, and 1 μg	Yes	No	Southern blotting qRT-PCR	6
Zaidan et al., 2019 (68)	HIV	HIV-infected CD4+ Cells	250–500	No	Yes	qRT-PCR	8

HBV, hepatitis B virus; EV71, enterovirus 71; HIV, human immune deficiency virus; HCV, hepatitis C virus; IAV, influenza A virus; VSV, vesicular stomatitis virus; HSV, herpes simplex virus; qRT-PCR, quantitative real-time polymerase chain reaction; ELISA, enzyme-linked immunosorbent assay; ECL, enhanced chemiluminescence; LDH, lactose dehydrogenase.

**Table 3:** Characteristics of studies included on IL-6.

Study	Viruses	Population	Dose (ng/mL)	Pre- infection treatment with cytokine	Co/Post-infection treatment with cytokine	Method of evaluation of viral titer	No. of replicates
Isorce et al., 2016 (69)	HBV	HepaRG cells PHH	1, 10, and 1,000	No	Yes	qRT-PCR	3
Hösel et al., 2009 (70)	HBV	PHHNPC HepG2.2.15	1, 5, 10, and 25	No	Yes	qRT-PCR	3
Kuo et al., 2009 (71)	HBV	HepG2 1.3ES2	0, 5, 10, 20, and 40	No	Yes	Southern blot Northern blot qRT-PCR	3
Zhu and Liu, 2003 (72)	HCV	FCA1 HUh7	0, 10, and 20	No	Yes	Northern blot analysis	3
Como et al., 2018 (73)	VZV	Primary induced pluripotent stem cells and primary human neuron cells	100	Yes	Yes	qRT-PCR Plaque assay	3
Moore et al., 2012 (74)	TMEV	B10.S and SJL/J mice, RAW264.7 cells, and macrophages	10	Yes	Yes	qRT-PCR	6
Danziger et al., 2018 (75)	HDV HIV MPV	LNCaP-JAK1 cells and BHK-21 ATCC CCL- 10 cells	5	Yes	Yes	Plaque assay Trypan Blue Exclusion assay FACS qRT-PCR	5

HBV, hepatitis B virus; HCV, hepatitis C virus; VZV, Varicella zoster virus; TMEV, Theiler’s murine encephalomyelitis virus; HDV, hepatitis D virus; HBV, hepatitis B virus; MPV, human metapneumovirus; FACS, fluorescence-activated cell sorting; qRT-PCR, quantitative real-time polymerase chain reaction.

**Table 4:** Characteristics of studies included on IL-22.

Study	Viruses	Population	Dose (ng/mL)	Pre- infection treatment with cytokine	Co/Post infection treatment with cytokine	Method of evaluation of viral titer	No. of replicates
Das et al., 2020 (76)	RSV	Primary human airway epithelial cells 2 and A549 cells	50	No	Yes	qRT-PCR Plaque assay	3
Yi et al., 2017 (24)	LCMV	C57BL/6 (B6) mice	IL-22–expressing plasmid: 10 μg	No	Yes	qRT-PCR	3
Xue et al., 2017 (77)	Rotavirus COVID PEDV COVID TGEV	IPEC-J2 cells, Vero E6 cells, and MA104 cells	Rotavirus: 40 COVID: 0, 4, 40, and 400	No	Yes	qRT-PCR Plaque assay	3
Schnepf et al., 2021 (78)	Rotavirus	C57BL/6J mice	1,000	Yes	No	qRT-PCR MicroArray	9

RSV, respiratory syncytial virus; LCMV, lymphocytic choriomeningitis virus; COVID, coronavirus disease; PEDV, porcine epidemic diarrhoea virus; TGEV, transmissible gastroenteritis virus; qRT-PCR, quantitative real-time polymerase chain reaction.

### Standard mean differences of IFN-λ, IL-32γ, IL-6, and IL-22

The analysis was carried out using the standardized mean difference as the outcome measure. A random-effects model was fitted to the data. Highest SMD was noted for IFN-λ with 0.9540, then IL-32γ with 0.459, IL-6 with 0.456, and IL-22 with 0.244 (**Table 5**). The average outcome differed significantly from zero for only IFN-λ and IL-32γ with z-score of 8.06 and 2.07 and p-values of <0.001 and 0.039, respectively, and, therefore, considered to be significant. The average outcome did not differ significantly from zero for IL-6 and IL-22 with z = 1.7978 and 0.8397, and p = 0.0722 and 0.4011, respectively.

**Table 5:** Meta-analysis of IFN-λ, IL-32γ, IL-6, and IL-22.

Cytokine	Studies	SMD	SE	z	p	CI lower bound	CI upper bound	Egger’s test (p-value)
IFN-λ	41	0.9540	0.134	7.13	<0.001	0.69	1.22	4.138 (<0.001)
IL-32γ	12	0.459	0.222	2.07	0.039	0.02	0.90	−0.622 (0.534)
IL-6	9	0.456	0.254	1.80	0.072	−0.04	0.95	−0.003 (0.998)
IL-22	6	0.244	0.291	0.840	0.401	−0.33	0.81	−0.521 (0.602)

P-value of <0.005 was considered statistically significant.

SMD, standard mean difference; SE, standard error; CI, confidence of interval.

### Forest plots and heterogeneity statistics of IFN-λ, IL-32γ, IL-6, and IL-22

**Figure 2:**
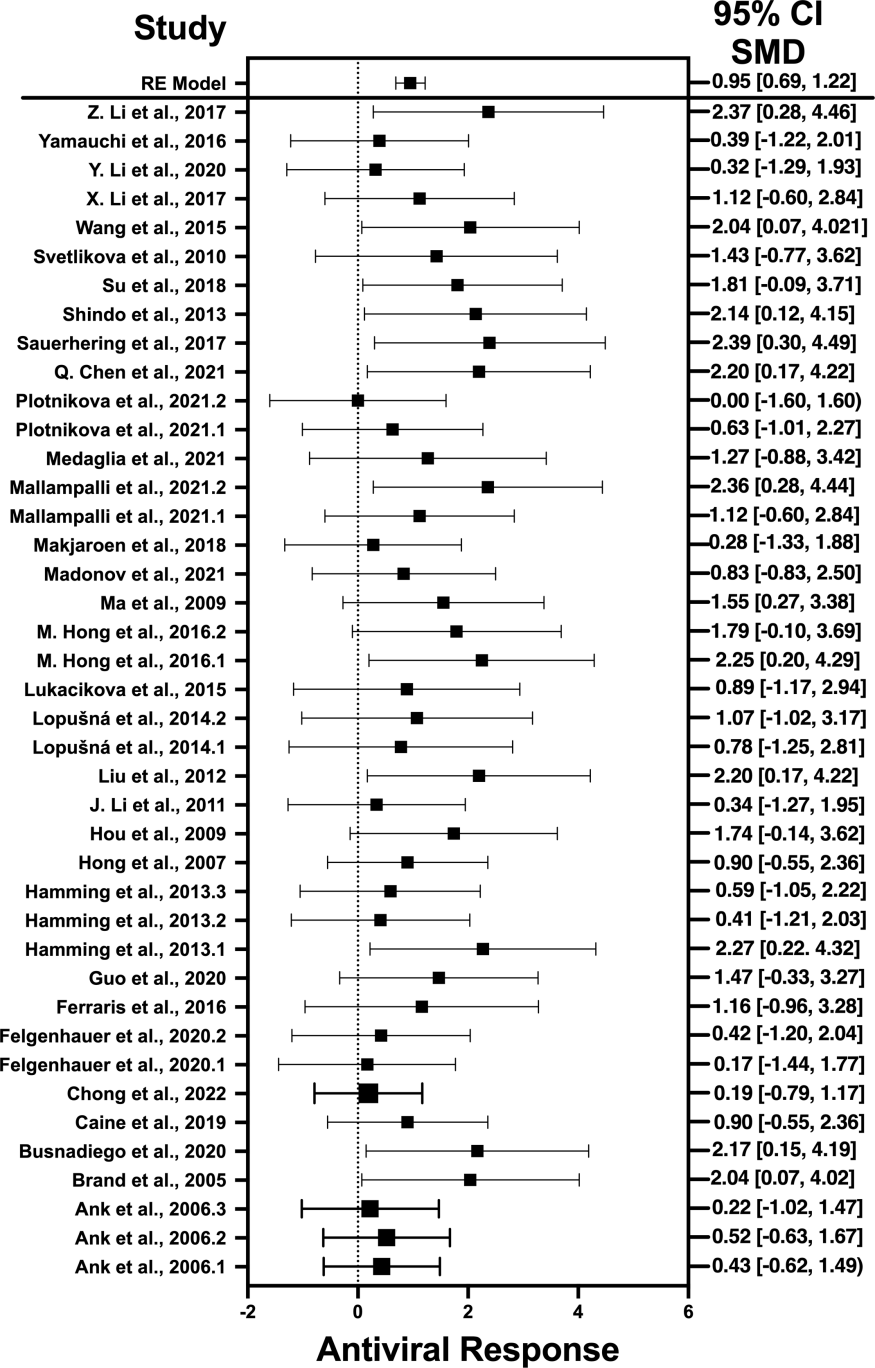
Forest plot illustrating antiviral response of IFN-λ according to the estimated effect sizes. The vertical line on the zero represents null hypothesis; the length of horizontal lines represents the total effect size of each study, starting with a lower limit and ending on an upper limit; and the squares are the mean differences. The antiviral effect IFN-λ is rated on a scale of 0 to 5. Square positioned above “0” is considered antiviral effect of IFN-λ, whereas a square lying below “0” will favor its proviral role.

**Figure 3:**
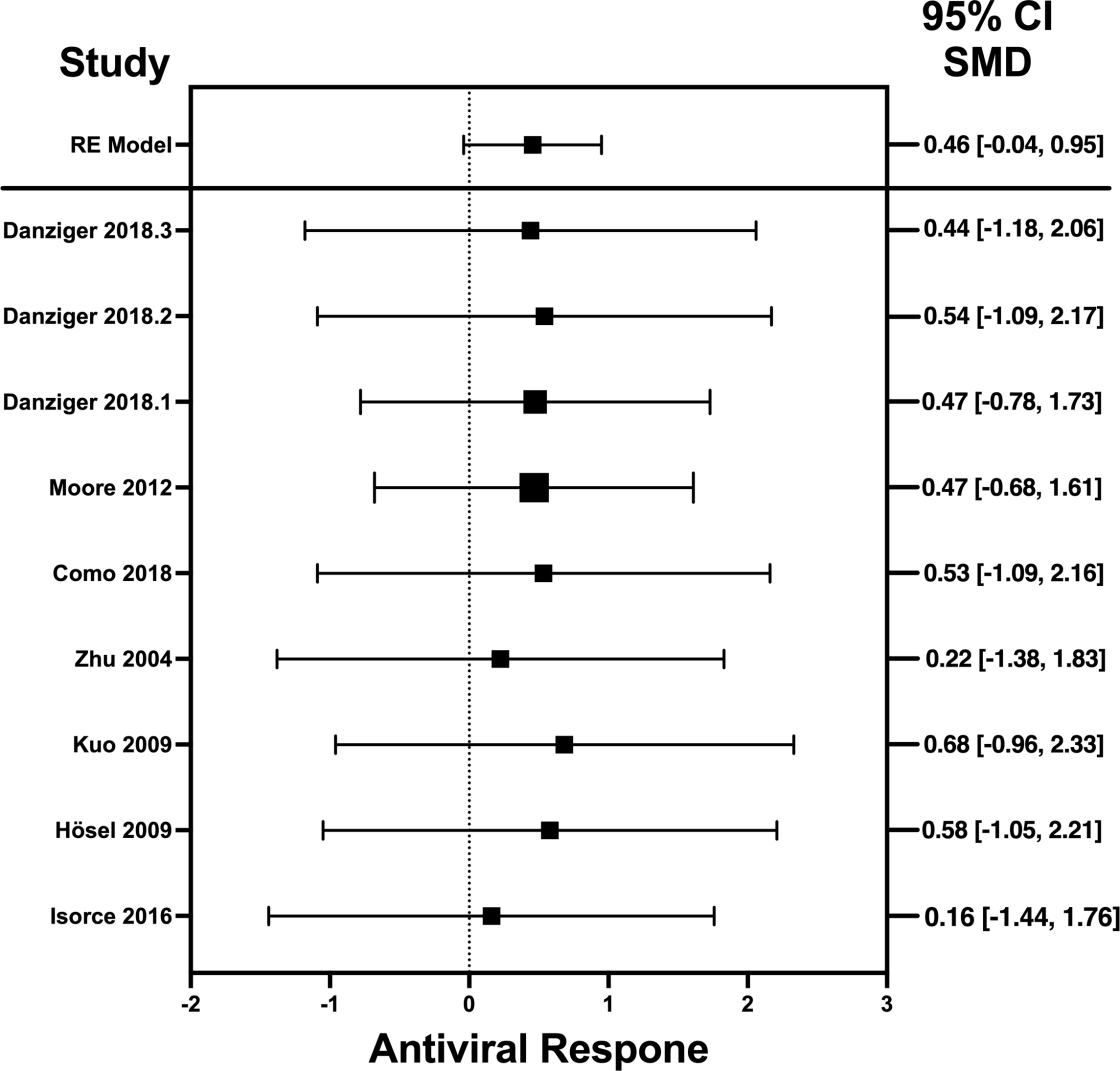
Forest plot illustrating antiviral response of IL-32γ according to the estimated effect sizes. The vertical line on the zero represents null hypothesis, the length of horizontal lines represents the total effect size of each study, starting with a lower limit and ending on an upper limit and the squares are the mean differences. The antiviral effect IL-32γ is rated on a scale of 0 to 5. Square positioned above “0” is considered antiviral effect of IL-32γ, whereas a square lying below “0” will favor its proviral role.

**Figure 4:**
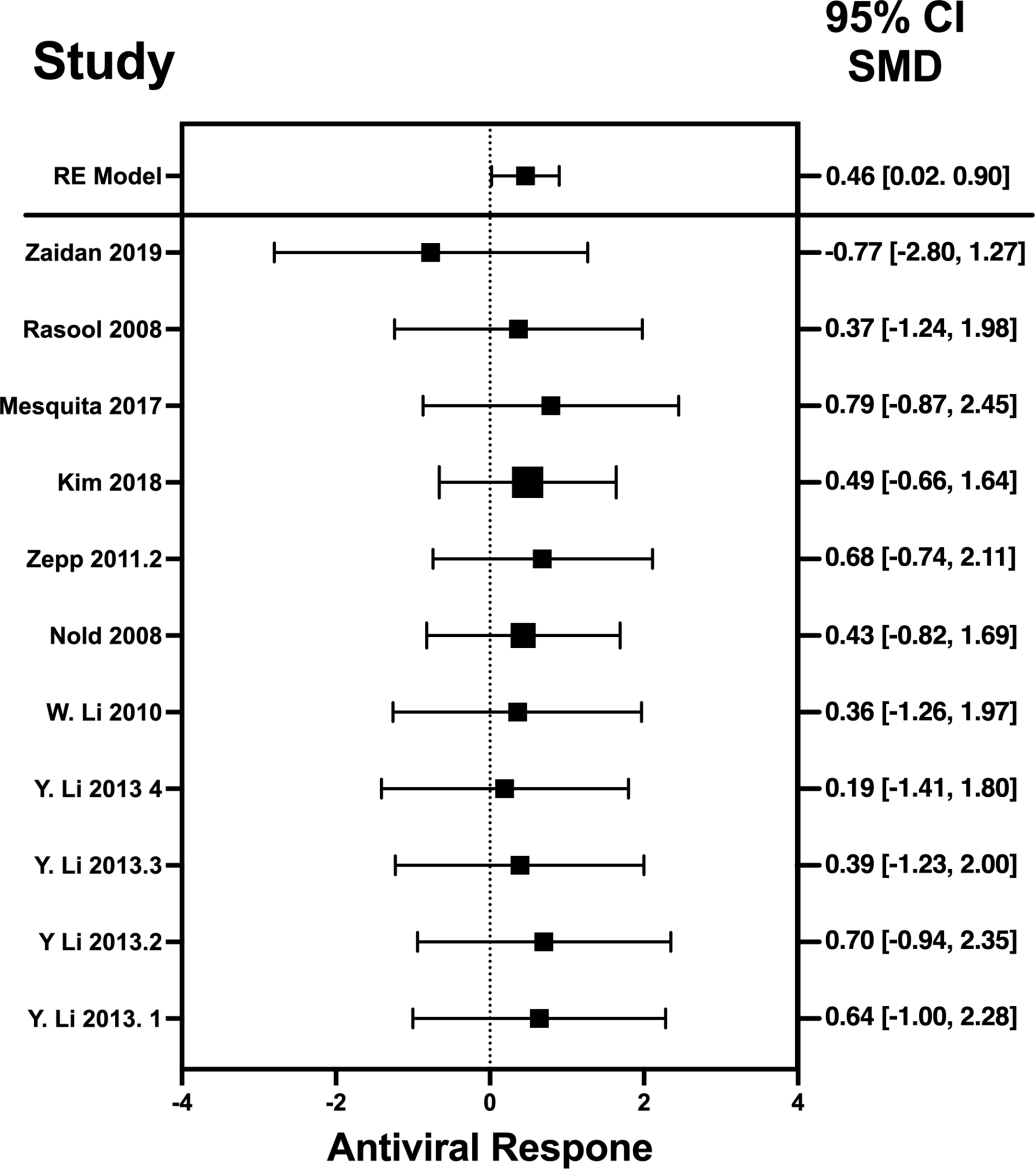
Forest plot illustrating antiviral response of IL-6 according to the estimated effect sizes. The vertical line on the zero represents null hypothesis; the length of horizontal lines represents the total effect size of each study, starting with a lower limit and ending on an upper limit; and the squares are the mean differences. The antiviral effect IL-6 is rated on a scale of 0 to 5. Square positioned above “0” is considered antiviral effect of IL-6, whereas a square lying below “0” will favor its proviral role. Square positioned above “0” is considered antiviral effect of IL-6, whereas a square lying below “0” will favor its proviral role.

**Figure 5:**
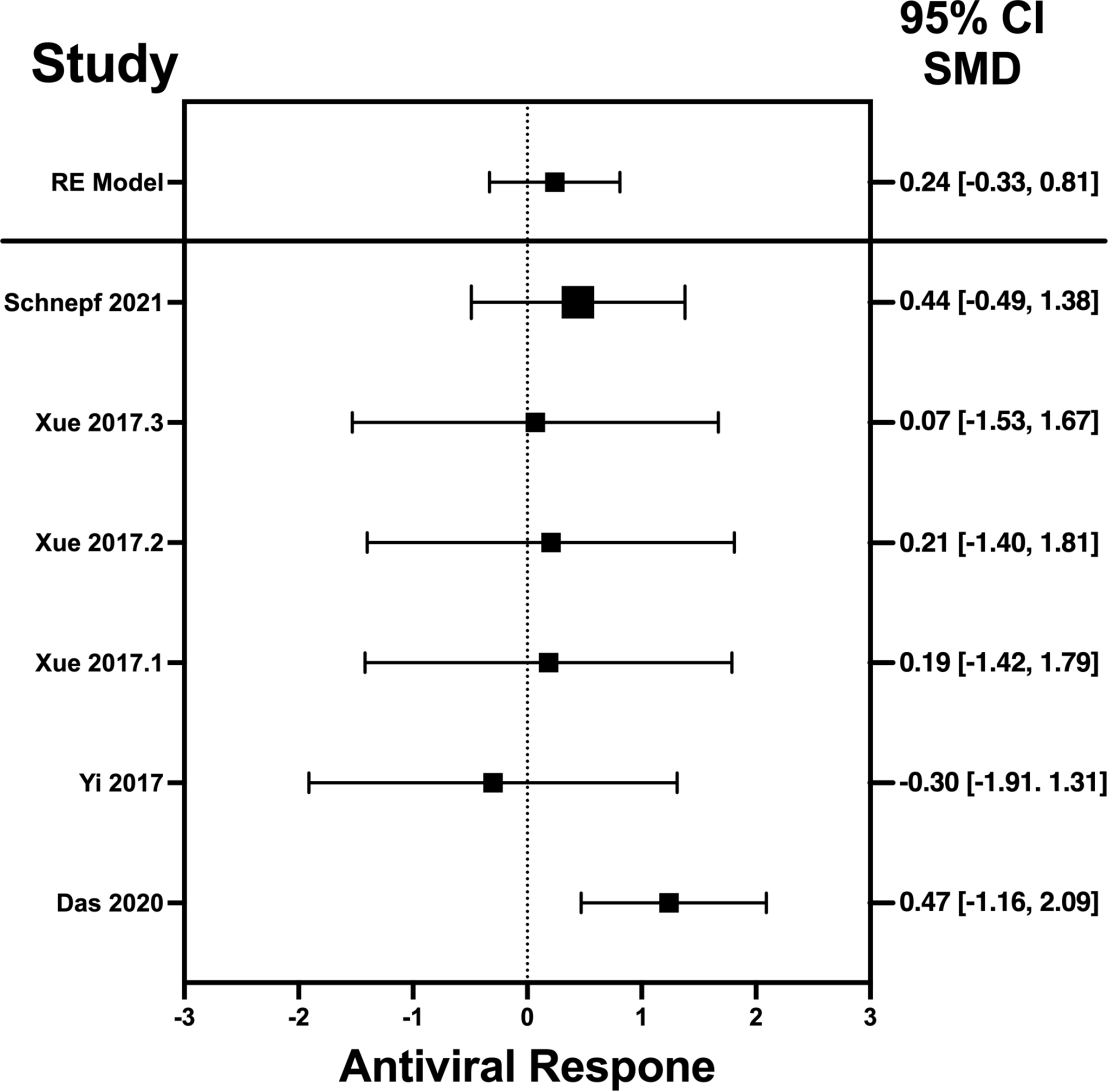
Forest plot illustrating antiviral response of IL-22 according to the estimated effect sizes. The vertical line on the zero represents null hypothesis; the length of horizontal lines represents the total effect size of each study, starting with a lower limit and ending on an upper limit; and the squares are the mean differences. The antiviral effect IL-22 is rated on a scale of 0 to 5. Square positioned above “0” is considered antiviral effect of IL-22, whereas a square lying below “0” will favor its proviral role. Square positioned above “0” is considered antiviral effect of IL-22, whereas a square lying below “0” will favor its proviral role.

Forest plots revealing statistical heterogeneity of estimated effects of the treatment in all the included studies are shown in **Figures 2**–**5**. The heterogeneity tests scores are mentioned in **Table 6** for all the four cytokines. None of four cytokines’ true outcomes appear to be heterogeneous (tau² < 0). The examination of the studentized residuals indicated no outlier data from any of the four cytokines. According to the Cook’s distances, none of the studies of IL-6 and IL-22 could be overly influential. However, study by Chong et al. (2022b) of IFN-λ and another study by Zaidan et al. (2019) of IL-32γ could be overly influential.

**Table 6:** Meta regression of IFN-λ, IL-32γ, IL-6, and IL-22.

Cytokine	Tau²	SE	I²	DF	Q	p
IFN-λ	0	0.1484	0%	40	30.599	0.858
IL-32γ	0	0.2456	0%	11	2.017	0.998
IL-6	0	0.2827	0%	8	0.330	1.000
IL-22	0	0.3178	0%	5	0.732	0.981

P-value of <0.005 was considered significance.

### Risk of publication bias

According to Egger’s linear regression test of IFN-λ, the funnel plot showed asymmetry with a p-value of 0.001. This may suggest that one or more studies included for IFN-λ carry risk of publication bias. Other than that, IL-32γ, IL-6, and IL-22 Egger’s test did not reveal any funnel plot asymmetry (p-values given in **Table 5**).

## Discussion

This systematic review and meta-analysis was conducted to aggregate data from multiple studies, revealing that cytokines such as IL-6, IL-22, IL-32γ, and IFN-λ have antiviral potential when administered exogenously. These pro-inflammatory cytokines regulate immune system communication and serve as mediators in the defense against viral pathogens. In addition to their well-known role in inflammation and immune regulation, cytokines are considered to exhibit direct antiviral activity (79).

To our knowledge, this is the inaugural study that systematically reviews existing evidence and performs a comprehensive meta-analysis to evaluate pro-inflammatory cytokines as alternative therapeutic agents to conventional antiviral drugs. Although various interleukins like IL-1β, IL-18, and TNF-α have been shown to inhibit viral infections (24), IL-6, IL-22, IL-32γ, and IFN-λ were selected due to high number of studies focusing on them.

The SMD measured for all the selected cytokines revealed that it was larger for IFN-λ compared to IL-6, IL-22, and IL-32γ. This high SMD indicates that IFN-λ has a more substantial antiviral impact than the other three interleukins. Then, a high number of investigations (please refer to **Table 1**) have been conducted on the IFN-λ’s antiviral activity, and, now, it is in phase 2 clinical trials as well (80).

The heterogeneity of the cytokines studied in this systematic review and meta-analysis was represented through Forest plots. As suggested and depicted by the Forest plots, the lack of variability in the antiviral activity of any of the four cytokines studied in this systematic review and meta-analysis suggests that the observed antiviral effects of the cytokines were more likely attributable to their true antiviral effects of these cytokines.

Results revealed no outliers or overly influential studies in the IL-6 and IL-22 datasets, although the SMDs of the two studies, Zaidan et al. (2019) (76) and Chong et al. (2022) (33) deviated from the average SMDs of all studies in the IL-32γ and IFN-λ datasets, respectively. In fact, the study by Zaidan et al. (2019) (68) indicated a proviral effect of IL-32, suggesting that the effect of IL-32 on viral infection is complex. It is possible that IL-32 has different effects on different viruses or that its effects vary depending on the specific population that is studied. Egger’s linear regression test was done to estimate the publication bias (81). The publication bias is checked through the depiction of asymmetry of the funnel plot generated as part of output of Egger’s regression. Among all cytokines studied, only the funnel plot of IFN-λ was asymmetrical regardless of zero heterogeneity. This asymmetry indicates publication bias, and one of the major reasons is the suppression of the null findings by the authors (82). Another possible reason for this asymmetry could be the use of the random-effects model (83).

IL-6, IL-22, IL-32, and IFN-λ are now being considered as potential allies in the struggle against viral infections. IFN-λ, also known as type III interferon, has demonstrated notable action against a variety of viruses, including respiratory viruses like influenza and SARS-CoV-2 (84). Being a pro-inflammatory cytokine, IL-6 has been shown to have antiviral effects against several viral infections (HBV, HCV, VZV, etc.) and is essential for immune responses. IL-22, another pro-inflammatory and tissue-protective cytokine, has shown antiviral activity against several viruses, including hepatitis C and the human papillomavirus. IL-32 has demonstrated antiviral potential by preventing viral replication and modifying host immune responses (85). These cytokines promise as possible antiviral therapeutics, whether used alone or in conjunction with already available antiviral medications.

Although several antiviral drugs are commercially available, they often come with side effects. These side effects could be reversible or mild like flu-like symptoms or can significantly cause the neurotoxic effects in patients (86). There is multiple evidence of scientific research in which scientists are using the combination of antiviral drugs with cytokines for best results. Chudhary et al. (2023) used the multiple antiviral drugs against HCV and concluded pegylated IFN + Ribavirin to be more effective (87).

Results indicate that, among the selected pro-inflammatory cytokines, IFN-λ has shown the best antiviral activity. Among the selected studies, Sauerhering et al. (2017) (67) has the highest effect size of 2.39 (CI: 0.30–4.49). This indicates its high antiviral activity against the Nipah virus, whereas IL-22 presents the lowest antiviral activity among the selected viruses. This is because IL-22 does not produce a direct antiviral response by interacting with the viral genes, instead it initiates the underlying signaling pathways that recruits the IFN-λ or cooperates with IL-18 to induce the interferon-stimulated gene (ISG) production and, hence, provides the protective role (88). Another restricting element is that not every pro-inflammatory cytokine generates the adaptive immune response to induce the viral suppression; instead, they generate the innate immune response to combat the viral infection (89). That is why the results of IL-22 was different from the other selected cytokines as it acts as a key modulator of innate immune response and is not directly involved in the adaptive immunity (90). Similar trend is observed when IL-32γ induces the antiviral responses against influenza virus (70), vesicular stomatitis virus (VSV) (91), and human immunodeficiency virus (HIV) (72). It produce IFN-λ that, in turn, suppresses the viral infection via generation of immune modulators (75). IL-6 not only produces the immune response against viruses but also interacts with certain viral genes that are responsible for the viral entry (92) and viral replication (78).

On the other hand, IFN-λ has superior antiviral activity than IL-6, IL-22, and IL-32γ. This is attributed to the induction of specific antiviral genes as well as the upregulation of ISGs. IFN-λ has been shown through *in vitro* experiments to specifically upregulate the antiviral genes in the epithelial cells. This results in the unique antiviral transcript profile and a strong induction of ISGs like MX Dynamin Like GTPase 2 (MX2), ISG15, and Interferon-induced protein with tetratricopeptide repeats 3 (IFIT3), which all contribute to the strong antiviral activity (93). Furthermore, IFN-λ offers strong defense at anatomical barriers such as epithelial surfaces and exhibits the notable antiviral activity against specific virus like EMCV, LCMV, and HSV (94).

This comprehensive systematic review and meta-analysis provides valuable insights. On the other hand, it has some limitations too, including the paucity of studies involving exogenously administered cytokines and the restricted range of cytokines tested for antiviral effects. This paucity regarding the availability of ample literature can be a major possible reason for the biasness of the reported data for this study. Another limiting factor of this study could be the evaluation of very few cytokines for their antiviral effect. To address these limitations, scientists should prioritize research involving the administration of recombinant cytokines or their expression vectors to provide a robust understanding of their antiviral effect in *in vitro* and *in vivo* settings as well. In addition, more cytokines should be explored for their potential antiviral properties.

In conclusion, this meta-analysis provides significant evidence supporting the potent antiviral properties of exogenous cytokines, specifically IL-6, IL-22, IL-32γ, and IFN-λ, against human and animal viral infections. Overall, these findings highlight the potential therapeutic applications of four important cytokines in the development of novel antiviral strategies, thus warranting further investigation to elucidate their precise mechanisms of action and optimize treatment protocols.

## Data availability statement

The original contributions presented in the study are included in the article/supplementary material. Further inquiries can be directed to the corresponding author.

## Author contributions

AS: Conceptualization, Data curation, Investigation, Methodology, Project administration, Software, Writing – original draft, Writing – review & editing. AQ: Conceptualization, Data curation, Investigation, Methodology, Project administration, Software, Writing – original draft, Writing – review & editing. SA: Data curation, Methodology, Writing – original draft. RM: Data curation, Methodology, Writing – original draft. HK: Data curation, Writing – original draft, Investigation. SM: Formal analysis, Supervision, Validation, Writing – review & editing. JA: Formal analysis, Validation, Writing – review & editing.
